# Little league shoulder revisited: a systematic review of the last decade

**DOI:** 10.1093/bmb/ldag044

**Published:** 2026-11-02

**Authors:** Annika Lenz, Luke Johnson, Mark Riederer, Aaron Provance

**Affiliations:** Department of Orthopaedics, University of Utah, 590 Wakara Way, Salt Lake City, UT 84108, United States; Department of Physical Medicine and Rehabilitation, University of Utah, 85 N. Medical Drive, Salt Lake City, UT 84132, United States; Department of Orthopaedics, University of Utah, 590 Wakara Way, Salt Lake City, UT 84108, United States; Department of Physical Medicine and Rehabilitation, University of Utah, 85 N. Medical Drive, Salt Lake City, UT 84132, United States; Department of Orthopaedics, University of Utah, 590 Wakara Way, Salt Lake City, UT 84108, United States; Department of Orthopaedics, University of Utah, 590 Wakara Way, Salt Lake City, UT 84108, United States; Department of Pediatrics, University of Utah, 295 Chipeta Way, Salt Lake City, UT 84108, United States

**Keywords:** Little League Shoulder, adolescent overhead athlete, systematic review, proximal humeral epiphysiolysis, overuse injuries

## Abstract

**Background:**

Physeal stress injuries involving the shoulder epiphyseal growth plate complex, known as Little League Shoulder (LLS), occur primarily in skeletally immature overhead athletes.

**Sources of data:**

This systematic review included research on LLS published within the last 10 years, from 1 January 2015 through 23 September 2025, from four electronic databases (Embase, PubMed, SPORTDiscus, Cochrane Library) focusing on diagnosis, management, imaging, and criteria for return-to-sport.

**Areas of agreement:**

LLS is mainly described in male, skeletally immature overhead athletes aged 11–15. Early specialization, multi-team participation, and inadequate rest are reported risk factors. LLS commonly presents with throwing-related shoulder pain, proximal humeral physis tenderness, posterior capsule tightness, and scapular dyskinesia. X-rays remain first-line imaging, demonstrating lateral physeal widening and irregularity. Throwing cessation for 2–4 months, followed by rehabilitation and gradual return-to-throwing yields excellent short-term outcomes, with low rates of growth disturbance. Prevention strategies include pitch-counts, rest, proper throwing mechanics, and delayed specialization.

**Areas of controversy:**

The prevalence of LLS remains unknown due to variable diagnostic criteria, imaging use, and underreporting. Recurrence and long-term sequelae are inconsistent across studies, and the reasons athletes discontinue baseball remain uncertain. Evidence is conflicting regarding the benefits of formal rehabilitation compared with rest alone.

**Growing points:**

Advanced imaging demonstrates structural remodeling of the shoulder, suggesting that repetitive loading during skeletal immaturity impacts long-term morphology and mechanics.

**Areas timely for developing research:**

Prospective studies using clinical and imaging criteria may help define the incidence, severity, risk factors, sport-specific patterns, and to clarify symptom resolution, humeral remodeling, and factors influencing attrition. Longitudinal studies should compare rest versus structured rehabilitation and varying return-to-throwing protocols.

## Introduction

Proximal humeral epiphysiolysis, commonly referred to as Little League Shoulder (LLS), continues to be a pervasive issue among skeletally immature overhead athletes, often due to early specialization and participation in year-round sports. With the increasing pressures to achieve high levels of success in sports, coaches and parents are pushing young athletes to practice at high intensity with inadequate rest, despite evidence demonstrating a link between early sports specialization and risk of injuries [1, 2]. Within baseball, youth and professional players experience high risks of upper extremity injuries, which has led to numerous organizations in the USA releasing injury prevention guidelines [3]. Although baseball players are the prototypical population due to the repetitive throwing motion and significant external rotational torque on the humeral shaft, LLS also occurs in other athletes, including tennis players and gymnasts.

Capturing the true incidence and prevalence of LLS remains challenging due to variations in diagnostic criteria and underreporting of symptoms. Contemporary evidence suggests that shoulder morbidity persists despite guideline implementation. Pennock *et al*. [4] reported that even among athletes who began participation after the introduction of Little League throwing guidelines and reported compliance with pitch count recommendations, a substantial proportion had a history of shoulder pain. These findings may suggest that guidelines are not consistently enforced and additional risk factors, such as early sport specialization and year-round participation, play a significant contributory role in the ongoing burden of injury. A 2024 scoping review of preventive measures for throwing injuries in baseball players suggested interventions such as sleeper stretching, shoulder external rotation strengthening exercises, and more comprehensive prevention programs while highlighting that age, sport level, and position of players may impact specific recommendations [5]. Although the presentation, risk factors, and management of LLS have been well documented without major changes recently, the lack of specific guidelines for diagnosis and treatment likely contributes to alterations in outcomes for these young athletes.

Over the last decade, advances in imaging, including the use of ultrasound (US) as a diagnostic modality and novel Magnetic Resonance Imaging (MRI) findings, have expanded our understanding of the injury beyond simple physeal widening. Additionally, new provocative stress tests to guide return-to-play decisions and emerging data on long-term sequelae such as persistent humeral retroversion have further challenged the adequacy of current treatment paradigms. The goal of this systematic review is to synthesize evidence from 2015 to 2025 to establish the prevalence and incidence of LLS, characterize injury severity, including time lost from sport and associated deformity, evaluate current approaches to diagnosis, treatment, and prevention, and critically examine whether the traditional definition of LLS adequately captures the full spectrum of proximal humeral physeal pathology.

## Materials and Methods

### Search strategy

This systematic review queried four electronic databases: Embase, PubMed, SPORTDiscus, and the Cochrane Library from 1 January 2015 through 23 September 2025. The search included the following terms: “Little League shoulder,” “Little Leaguer's shoulder,” “proximal humeral epiphysiolysis,” “proximal humeral physeal injury,” “proximal humeral physis stress injury,” “osteochondrosis of the proximal humeral epiphysis,” “osteochondrosis of the proximal humerus,” “chronic overuse osteochondritis,” “adolescent athlete’s shoulder,” “overuse injury of the proximal humeral growth plate,” “stress injury of the proximal humeral growth plate,” “Salter-Harris type 1 displacement of the physis,” “type 1 Salter-Harris stress fracture of the proximal humerus,” “slipped capital humeral epiphysis,” and “proximal humeral epiphysitis.” The terms “epidemiology,” “injury rate,” and “injury prevention” were not explicitly included in the search. However, relevant epidemiologic and incidence-related outcomes were captured using broader LLS-related search terms.

The search was further refined to include only articles published within the last 10 years that contained these terms in the title or abstract. All citations were imported into Covidence [6] for screening.

### Inclusion and exclusion criteria

Articles were included if they were novel research papers, case studies, case series, review articles, prospective and retrospective cohorts and if the study population included individuals aged 8–19. Articles were excluded if they involved acute fractures or traumatic injuries to the proximal humeral physis, acute shoulder injuries, anatomical locations other than the proximal humeral physis/epiphysis, surgical cases, non-English languages, published before 2015, or included animal models.

### Study screening

The initial search provided 78 articles, of which 29 were duplicates. Two reviewers (A.L. and L.J.) independently screened the titles and abstracts of the remaining 49 articles. Conflicts were resolved by a senior reviewer (M.R.). A total of 33 articles underwent full-text review. These articles were divided amongst the reviewers and assessed independently. Then, the group met to discuss any studies that did not meet eligibility criteria. Sixteen studies were excluded at this stage due to wrong study design (*n* = 13), wrong patient population (*n* = 2), and wrong outcomes (*n* = 1). A total of 17 articles met eligibility criteria and were included in the final analysis.

### Data extraction

Data from all included studies were extracted into a customized Excel spreadsheet. Recorded variables included publication details, manuscript type, manuscript purpose, level of evidence, population, intervention, physical therapy prescription, interval throwing program prescription, outcome, and a brief narrative summary of each study. Results were synthesized using a narrative summary approach.

### Quality assessment

Study quality was assessed using the Methodological Index for Non-Randomized Studies (MINORS) [7]. Each item was scored from 0 to 2, with a maximum score of 16 for non-comparative studies and 24 for comparative studies. Review articles were not included in the quality assessment as MINORS is designed to evaluate non-randomized studies and is not applicable to review articles. Non-comparative studies were categorized as low quality (0–7), moderate quality (8–11), or high quality (12–16). Comparative studies were categorized as low quality (0–11), moderate quality (12–17), or high quality (18–24). Studies with higher scores represent stronger quality and lower risk of bias.

## Results

This review followed the PRISMA (Preferred Reporting Items for Systematic Reviews and Meta-Analyses) guidelines [8] (Fig. 1). Of the 17 studies meeting the inclusion criteria in this systematic review, six were retrospective cohort or case–control studies, eight were case reports or case series, and three were review articles (Table 1). With most articles being retrospective observational studies or case reports (level 3 or 4 evidence), causal associations are limited and the studies are subject to selection bias. Additionally, two studies scored in the low-quality category using the MINORS checklist, reflecting low methodological quality and a higher risk of bias [7].

**Figure 1:**
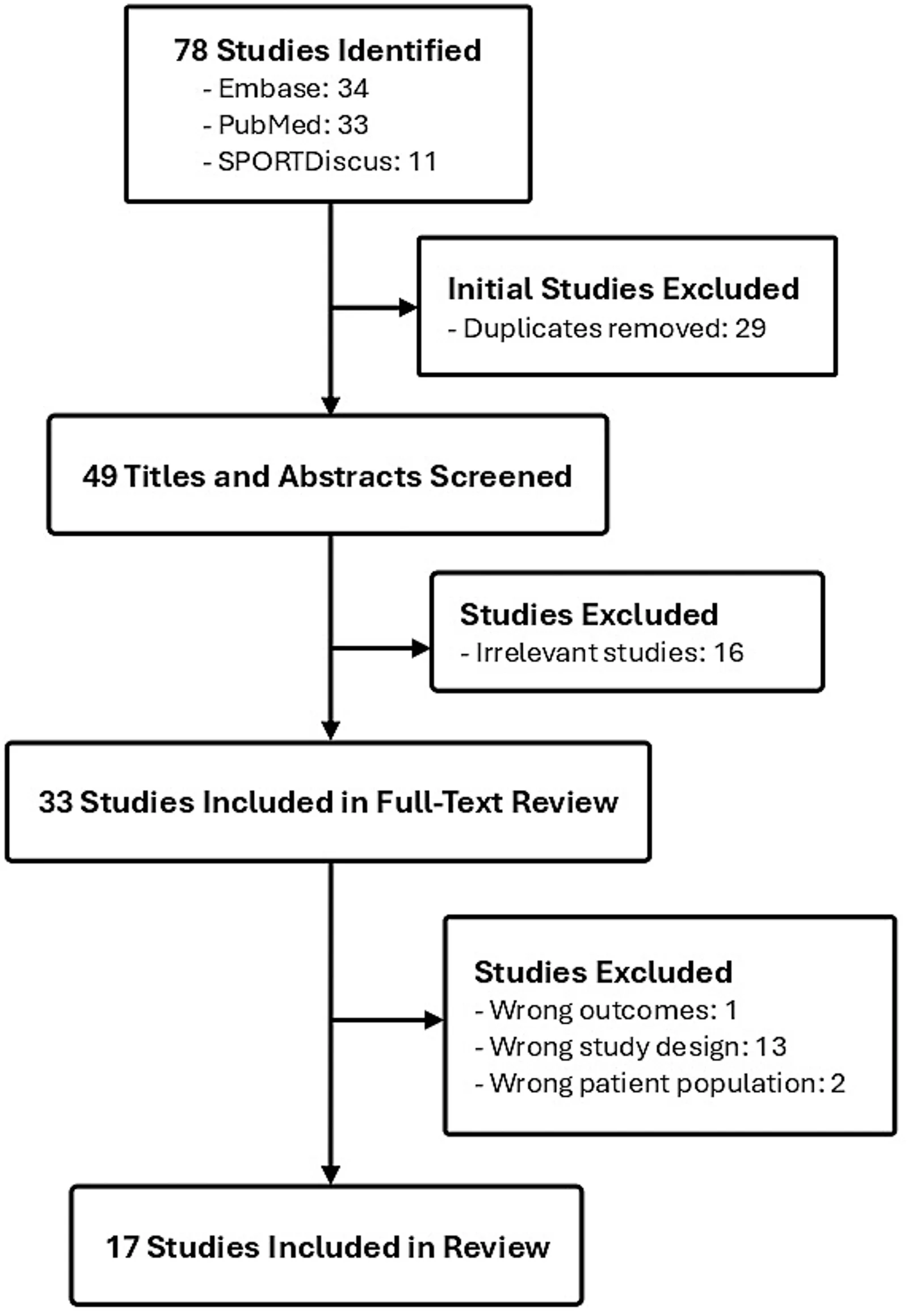
PRISMA flow chart on systematic review on LLS.

**Table 1:** LLS study characteristics.

Lead author (year)	Country of publication	Study design (LOE)	MINORS score (out of 16 for non-comparative and 24 for comparative)	Sample size	Girls %	Age, yo	Injury frequency
Bedoya *et al*. (2015) [9]	USA	Narrative review (IV)	n/a	n/a	n/a	11–16 yo	NR
Reeder and Smith (2015) [10]	USA	Case report (IV)	7	1	0%	13 yo	NR
Heyworth *et al*. (2016) [11]	USA	Case series (IV)	10	95	2%	8–16 yo	95 patients diagnosed with LLS between 1999 and 2013
Hosokawa *et al*. (2017) [12]	Japan	Case report (IV)	9	1	0%	15 yo	NR
Greenberg *et al*. (2018) [13]	England	Case report (IV)	9	1	0%	15 yo	NR
Harada *et al*. (2018) [14]	Japan	Retrospective cohort (II)	9	87	NR	7–17 yo	87 baseball players with LLS
Pennock *et al*. (2018) [4]	USA	Case–control study (III)	21 (comparative)	23	0%	10–12 yo	22% (*n* = 5) of players had MRI findings indicating LLS
Zipser *et al*. (2018) [15]	USA	Case report (IV)	7	1	0%	15 yo	NR
Ito *et al*. (2019) [16]	USA	Cohort study (III)	21 (comparative)	10	0%	11–18 yo	10 baseball players with past LLS diagnosis
Bednar *et al*. (2021) [17]	USA	Systematic review (IV)	n/a	n/a	n/a	11–16 yo	266 participants diagnosed with LLS across 23 studies
Fehr *et al*. (2021) [18]	Germany	Cohort study (III)	11	10	0%	12–16 yo	10 male baseball players with LLS
Jensen *et al*. (2024) [19]	USA	Retrospective cohort study (IV)	9	17	1%	9–17 yo	17 baseball and softball players with LLS
Myers *et al*. (2024) [20]	USA	Narrative review and case report (IV)	n/a	n/a	n/a	13 yo	NR
Omodani *et al*. (2024) [21]	USA	Two-patient case report (IV)	9	2	0%	13 and 15 yo	NR
Casadei and Kiel (2025) [22]	USA	Narrative review (IV)	n/a	n/a	n/a	11–16 yo	NR
Fortney *et al*. (2025) [23]	USA	Retrospective descriptive study (III)	11	75	0%	17–21 yo	Pitchers: 18.2% showed radiographic evidence of LLS Position players: 14.3% showed radiographic evidence of LLS
Uchino *et al*. (2025) [24]	USA	Case series (IV)	13	101	2%	7–17 yo	101 patients diagnosed with LLS

NR: Not reported.

### Epidemiology

Although the true prevalence of LLS in skeletally immature individuals is difficult to quantify in part due to nonuniform diagnostic criteria and underreporting, LLS represents a significant population-level burden. With more than 5 million children participating in youth baseball as of 2023 [25] and estimates of 30% of youth baseball players experiencing shoulder pain within a single season [20], this translates into a large number of shoulder overuse injuries overall. Given study design limitations, true incidence data cannot be determined. Furthermore, none of the studies included in this review reported injury rates of LLS; thus, injury frequency is included (Table 1). Heyworth *et al*. [11] highlighted the rising trend of LLS diagnoses from 1999 to 2013 with an average annual increase of 8.5%, which has been attributed to early sports specialization with year-round participation and increased pitching velocity. Although this diagnosis is often seen in pitchers, Fortney *et al*. [23] reported that 18.2% of pitchers and 14.3% of position players evaluated at a Major League Baseball scouting event had a current or prior diagnosis of LLS, suggesting its prevalence among all baseball players. Pennock *et al*. [4] found that 100% of the single-sport specialized baseball athletes participating in their study had abnormal MRI findings including physeal changes.

The prognosis of LLS tends to be favorable overall with few cases developing severe complications. Rates of return to sport following rest and rehabilitation ranged from 81% to 100% according to Harada *et al*. [14]. Bednar *et al*. [17] reported that 94% of athletes returned to sport, with 92.5% returning to their preinjury level. However, recurrence remains a concern, with rates ranging from 7% to 25% in the months following return to sport and up to 41% at 5-year follow-up [12, 14].

Additionally, persistent arm pain contributes to athletes quitting baseball earlier than intended. In Jensen’s study looking at the long-term prognosis of baseball players with LLS, 39% of the baseball players who quit baseball reported it was due to arm pain [17]. Serious complications such as premature physeal closure leading to growth disturbance are exceedingly rare. Bednar *et al*. [17] reported only one out of 266 athletes developed premature closure of the physis. Despite the rarity of growth disturbance issues, the physeal stress may be associated with skeletal adaptations such as increased humeral retroversion and varus deformity that alter shoulder biomechanics and increase future injury risk [12, 16].

### Demographics

Across the included studies, LLS commonly affects adolescent male athletes participating in overhead sports, particularly baseball (Table 1). The typical age at diagnosis ranged from 11 to 15 years, corresponding to skeletal immaturity of the proximal humeral physis [11, 14, 16, 19]. Reported mean ages include 12.1 years [14], 13.1 years [11], and 13.2 years [19], with Bednar’s [17] systematic review demonstrating a weighted mean age of 12.8 years across 266 athletes.

Male baseball athletes comprised nearly all affected individuals in every study, reflecting both sport participation patterns and true epidemiology [13, 16, 18, 20, 21], while Heyworth *et al*. [11] identified two female athletes, both of whom were baseball pitchers (Table 1). Harada *et al*. [14], Heyworth *et al*. [11], Uchino *et al*. [24], Jensen *et al*. [19], Ito *et al*. [16], and Fehr *et al*. [18] highlighted that pitchers consistently represented a high-risk subgroup. Additionally, Jensen *et al*. [19] found that about 80% of their LLS group played baseball more than 9 months per year while Ito *et al*. [16] noted that all players began baseball between ages 6 and 10 and participated year-round.

Although baseball predominates, similar proximal humeral physeal stress injuries have been described in other overhead or weight-bearing sports, including tennis, badminton, basketball, cricket, track, American football, softball, volleyball, and swimming [17]. Omodani *et al*. [21] described two gymnasts who developed symptoms of LLS during activities such as handstands, suggesting that repetitive upper-extremity loading rather than sport alone is the key mechanism.

Geographically, most studies originated from the USA, but international representation was notable in the broader literature (Table 1). Bednar *et al*.’s [17] systematic review included research from Japan, Canada, Germany, and the UK, while Fortney *et al*. [23] analyzed baseball players in the Dominican Republic, where formal injury prevention guidelines are not widely implemented.

### Physical examination and symptom presentation

Athletes with LLS typically present with activity-related shoulder pain localized to the dominant proximal humerus that is exacerbated by throwing or repetitive overhead activity [17]. There was not a single throwing phase consistently identified as the source of symptoms. Bednar *et al*. [17] reported both acute and insidious symptom onset, with duration ranging from 2 weeks to 1 year. Early symptoms were frequently underreported as many athletes felt that symptoms were initially perceived as manageable without intervention [20].

Heyworth *et al*. [11] reported that all athletes experienced pain with throwing, while a subset reported shoulder weakness or fatigue (10%), mechanical symptoms (8%), and subjective instability (1%). No neurologic complaints were identified. Elbow pain was documented in 13% of cases, often representing concurrent pathology, consistent with observations from Fortney *et al*. [23] and Casadei and Kiel [22]. Casadei and Kiel [22] also noted progressive onset of shoulder pain, occasional pain at rest in advanced cases, and decreased throwing velocity or accuracy, with recent growth acceleration or prior elbow symptoms as potential risk modifiers.

Physical examination findings were consistent across studies. Focal tenderness over the proximal humeral physis represented the most common clinical finding, reported in 74% of athletes in the Heyworth [11] cohort. Decreased shoulder range of motion was observed in 34% of cases, while 11% demonstrated shoulder girdle weakness and 30% exhibited glenohumeral internal rotation deficit (GIRD), although specific quantification of rotational deficits varied widely between studies.

More detailed biomechanical and structural findings were described in some case series. Greenberg *et al*. [13] documented bilateral alterations in glenohumeral rotational arc over a 2-year interval, with a 5° reduction in dominant-arm rotation and declines in humeral retrotorsion. Myers *et al*. [20] emphasized the frequent presence of scapular dyskinesis, posterior shoulder tightness, and periscapular or rotator cuff weakness. Hosokawa *et al*. [12] identified significant humeral morphological alterations in a previously diagnosed patient, including increased retroversion, varus deformity, and extension deformity relative to the contralateral side. Harada *et al*. [14] reported markedly limited shoulder flexibility among affected athletes. Ito *et al*. [16] quantified humeral retroversion using US, demonstrating significantly greater retroversion in the LLS group compared with controls.

### Imaging findings

Although LLS is primarily a clinical diagnosis, imaging plays a central role in confirming pathology, evaluating severity, and identifying alternative or concurrent injuries. Standard internal and external rotation anteroposterior radiographs are the first-line modality and remain the cornerstone of diagnosis [20]. Typical findings include widening, irregularity, sclerosis, or demineralization of the lateral aspect of the proximal humeral physis [11, 14, 19] (Fig. 2). Metaphysis fragmentation near the lateral physis may also be present when compared to the nondominant shoulder [20]. Modified axillary views may improve visualization of the posterior glenoid when posterior shoulder pathology is suspected [20].

**Figure 2:**
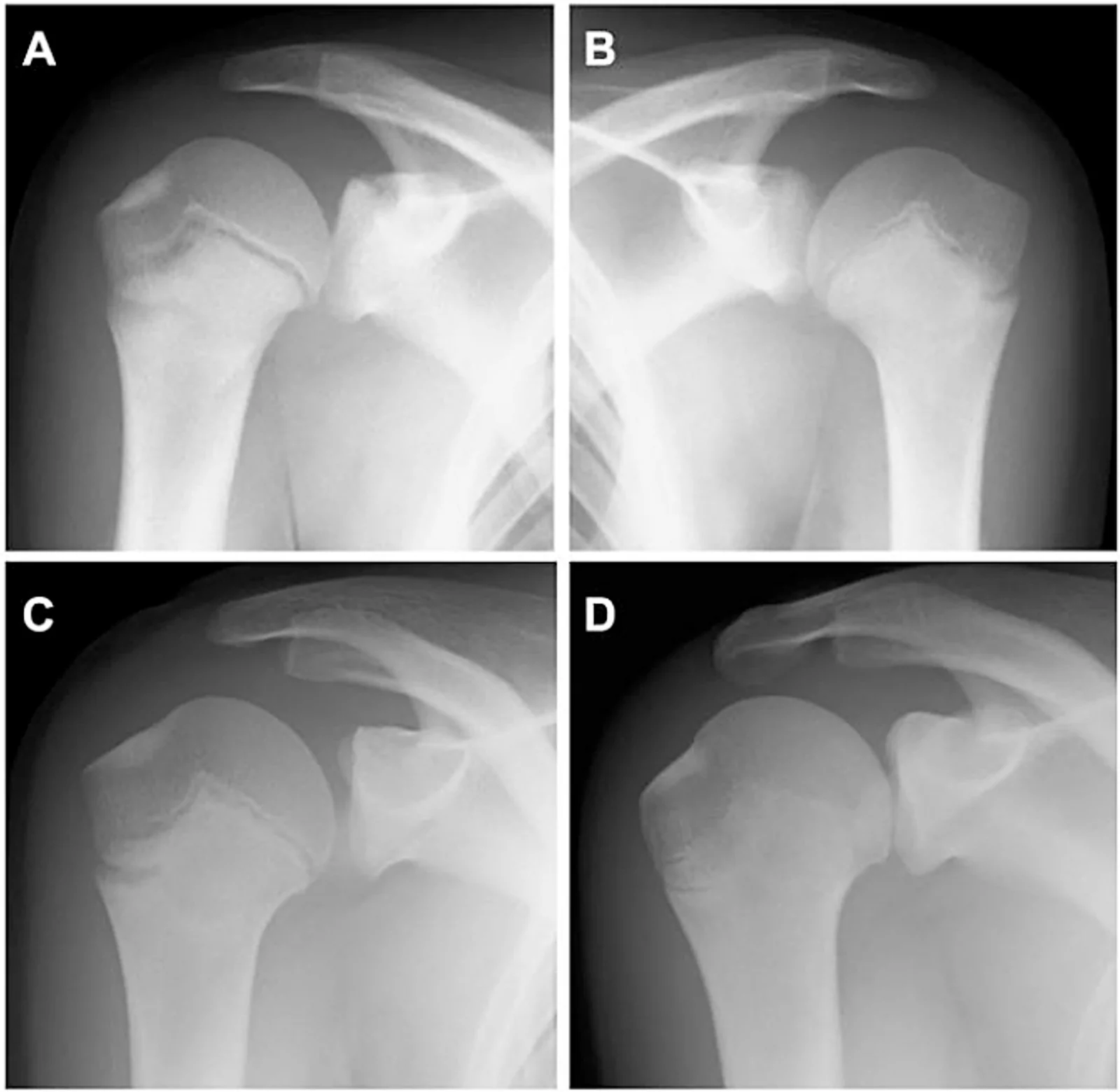
Four radiographic findings of LLS by Ito *et al*. [16] illustrating the progression of Little League Shoulder in a 12-year-old patient and subsequent healing; (A) widening of the proximal humeral physis on the dominant side at age 12 years, consistent with physeal stress injury, compared with (B) a normal-appearing proximal humerus on the nondominant side at the same age; after a 3-month period of rest from throwing, (C) repeat imaging showed normalization of the proximal physis, and the patient was able to resume throwing without pain; at long-term follow-up, (D) radiographs obtained 4 years after nonoperative treatment demonstrated appropriate closure of the proximal humeral physis by age 16 years.

In Heyworth *et al*.’s [11] comprehensive evaluation of LLS at a high-volume regional children’s hospital over 14 years, radiographs were obtained in 94% of patients and confirmed the diagnosis in 76%. Among those with positive radiographs, 11% underwent MRI, which confirmed LLS in all cases. Of patients with negative radiographs, five underwent MRI and all were positive. Overall, 17% were diagnosed clinically when imaging was negative or unavailable, and 3% were diagnosed by MRI alone.

MRI can be useful when radiographs are normal but clinical suspicion remains high. MRI may show physeal widening, early stress changes, metaphyseal and periosteal edema, subchondral bone marrow signal abnormalities, and physeal cartilage irregularity [9]. Bedoya *et al*. [9] noted that acute injuries typically lack physeal irregularity and present with more prominent soft-tissue edema, a distinction best identified with MRI.

Adjunct imaging modalities have expanded understanding of LLS-associated structural adaptations. Three-dimensional computed tomography (CT) analyses by Hosokawa *et al*. [12] demonstrated measurable valgus–varus, flexion–extension, and rotational deformities when comparing dominant and nondominant humeri (Fig. 3). Significant differences in tuberosity morphology and proximal humeral shape suggest that chronic physeal stress may lead to remodeling beyond retroversion increases. Similarly, Omodani *et al*. [21] described deformity on full-limb, handstand radiographs in a gymnast, noting decreased palm-to-glenohumeral distance and compensatory scapular abduction and elevation (Fig. 4).

**Figure 3:**
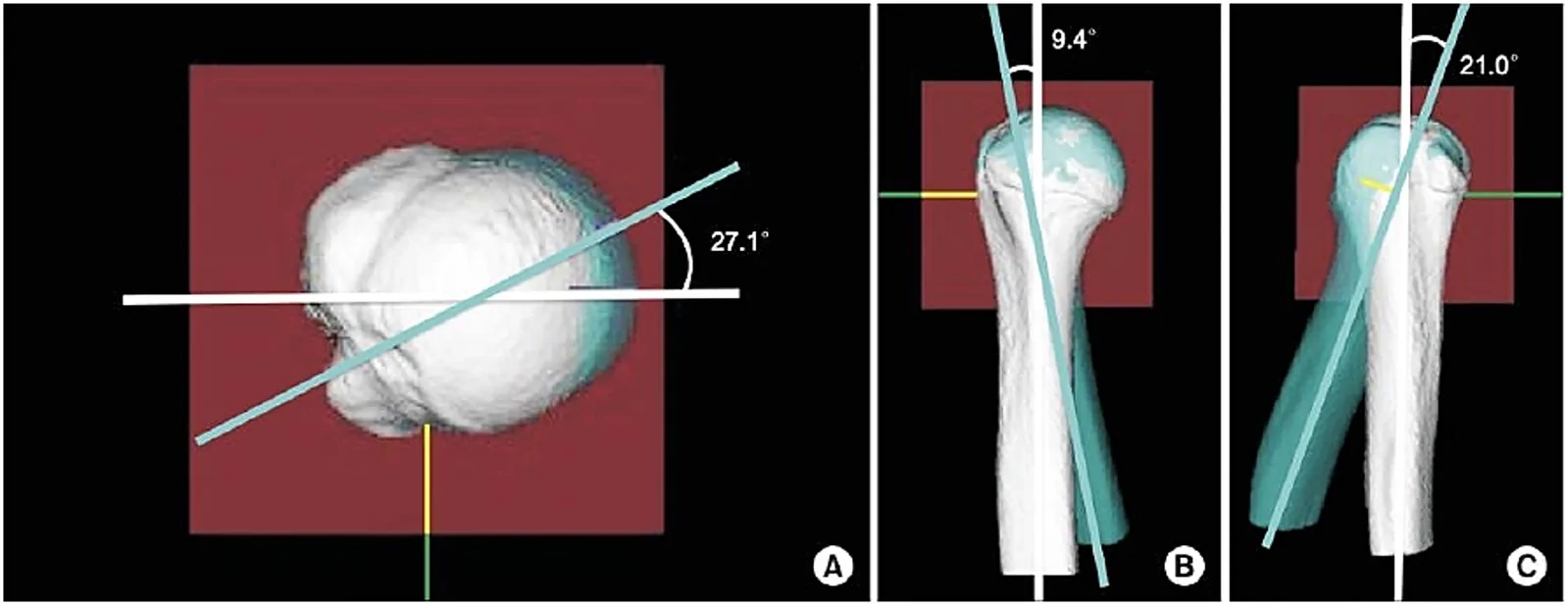
Three-dimensional CT reconstructions of the dominant and nondominant humeri by Hosokawa *et al*. [12] are shown side by side to compare bone shape and alignment. The images illustrate differences in the angle of the humerus in the coronal plane (valgus–varus), sagittal plane (flexion–extension), and axial plane (rotation), demonstrating asymmetrical bone alignment between the throwing and non-throwing arms.

**Figure 4:**
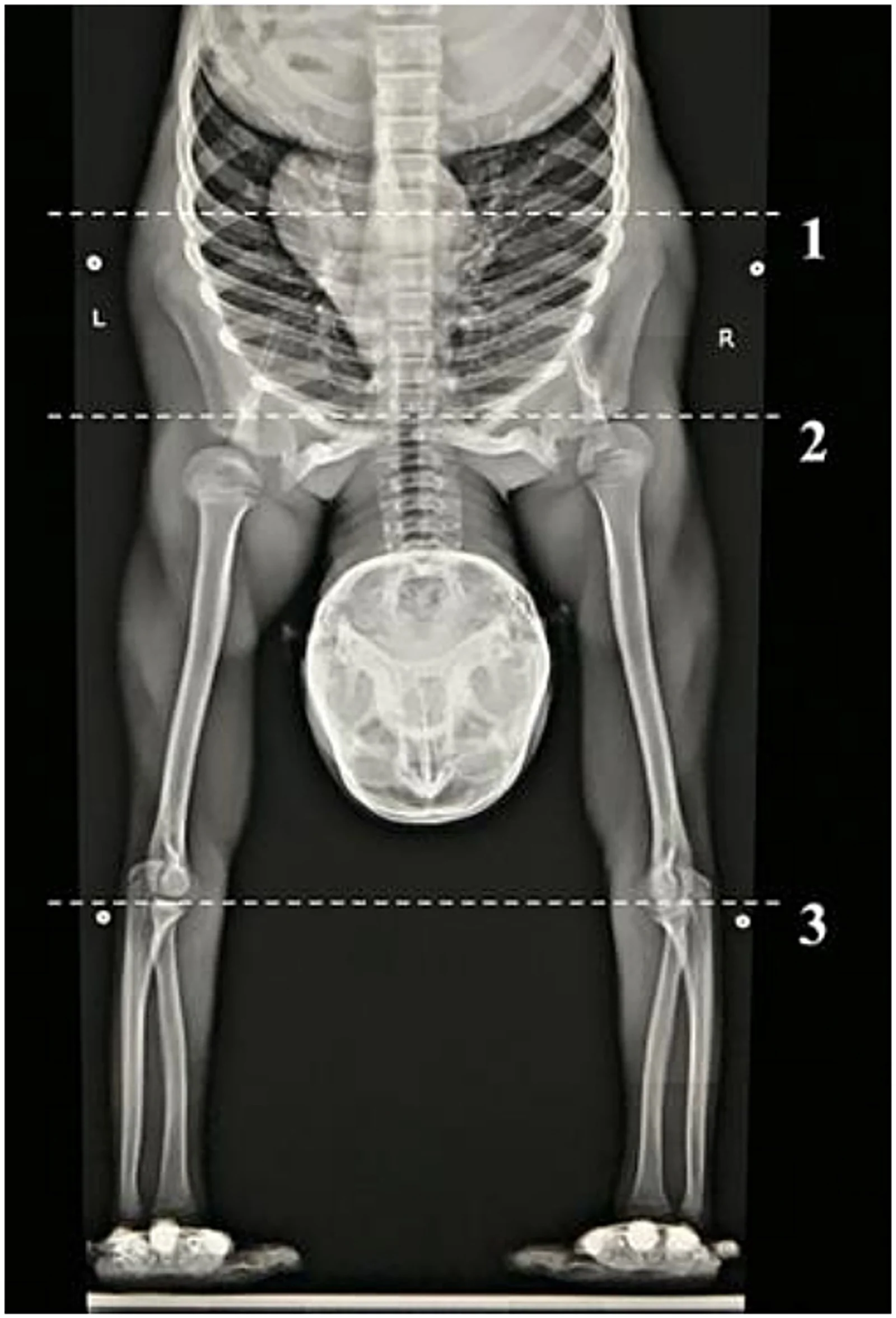
Radiograph of a gymnast in a handstand position, with the arms supporting the body weight overhead. The images show a decreased distance between the palms and the glenohumeral joints, indicating altered upper-extremity alignment, along with compensatory scapular abduction and elevation. Three lines are drawn horizontally on the radiographs to demonstrate the shortening of the humeral length with the compensatory movement of the scapula. Line 1: Height of the distal edge of the healthy side's scapula. Line 2: Height of the glenohumeral joint of the healthy side. Line 3: Height of the radioulnar joints on both sides Omodani *et al*. [21].

US has emerged as a valuable supplemental imaging tool. Casadei and Kiel [22] reported that hypoechoic swelling around the affected physis may be appreciated on US. Reliable sonographic methods for measuring humeral retroversion achieved high intraobserver and interobserver reliability. Fehr *et al*. [18] demonstrated similarly strong interrater reliability for proximal humeral physeal width measurements and found significant differences between dominant and nondominant shoulders. However, radiographs remain preferred when diagnostic uncertainty is high due to US’s inability to detect some clinically significant alternative conditions [12].

### Treatment and outcomes

Conservative, non-operative management was consistently described as the primary and most effective treatment strategy for LLS [11]. Standard protocols involved cessation of throwing or overhead loading, followed by structured, symptom-guided rehabilitation, and a staged return-to-throwing progression (Table 2). Outcomes associated with these approaches were overwhelmingly favorable, though long-term sequelae were variably reported.

**Table 2:** Management of LLS with physical therapy and interval throwing programs.

Lead author (year)	Was PT prescribed?	Was an interval throwing program prescribed?
Bedoya *et al*. (2015) [9]	NR	NR
Reeder and Smith (2015) [10]	Yes, thoracic mobilization	Yes, not specified
Heyworth *et al*. (2016) [11]	Formal physical therapy programs (*n* = 75)	NR
Hosokawa *et al*. (2017) [12]	NR	NR
Greenberg *et al*. (2018) [13]	Range of motion and humeral retroversion measurements	NR
Harada *et al*. (2018) [14]	1-month of rehabilitation aimed at improving shoulder tightness	NR
Pennock *et al*. (2018) [4]	NR	NR
Zipser *et al*. (2018) [15]	NR	NR
Ito *et al*. (2019) [16]	NR	NR
Bednar *et al*. (2021) [17]	Formal physical therapy (*n* = 4) At-home rehabilitation (*n* = 1)	Regimented throwing program (*n* = 1)
Fehr *et al*. (2021) [18]	NR	NR
Jensen *et al*. (2024) [19]	Offered but left to the family's discretion to pursue	Yes, a regimented throwing program is prescribed at 6 weeks
Myers *et al*. (2024) [20]	Formal physical therapy targeting deficits noted from objective measures, as well as patient education on throwing volume and its relation to injury risk	Individualized interval throwing program before returning to prior level of competition and throwing volume
Omodani *et al*. (2024) [21]	Case 1: No Case 2: Yes, treatments aimed at improving range of motion and strengthening the core	Case 1: No Case 2: No
Casadei and Kiel (2025) [22]	Treatments to strengthen core muscles and rotator cuff	Gradual return to throwing with a structured throwing program
Fortney i. (2025) [23]	NR	NR
Uchino *et al*. (2025) [24]	Stretching exercises aimed to improve the tightness of the shoulder, trunk, and lower extremity	Yes, a gradual increase in throwing intensity based on an established 1-month throwing protocol

NR: not reported.

Initial rest was the cornerstone of treatment. Heyworth *et al*. [11] recommended cessation of throwing in 94 of 95 athletes, prescribing an average of 4.2 months of rest. Athletes demonstrated symptom resolution in ~2.6 months and returned to competition by around 4.2 months, with no significant differences between those who engaged in formal rehabilitation and those who did not. Harada *et al*. [14] similarly emphasized early rest and restoration of flexibility, reporting complete resolution in their cohort of 87 players. Delayed initiation of rest and persistent pain at 1 and 2 months strongly predicted poor outcomes, with shoulder pain at 2 months increasing recurrence risk more than 10-fold [14].

Other studies reported shorter rest periods with similarly successful outcomes. For example, Ito *et al*. [16] found that 2–3 months of rest was sufficient to achieve radiographic healing and full return-to-play without formal physical therapy (Fig. 2). Hosokawa *et al*. [12] likewise described complete symptom resolution and full activity after 2 months of rest. Similar success was documented by Reeder and Smith [10] and Zipser *et al*. [15], with complete resolution and return-to-throwing within 3 months despite acute or dramatic presentations. Myers *et al*. [20] reported on an athlete with concomitant posterior glenoid dysplasia who required up to 6 months of rest, guided by serial radiographs before progressing to a kinetic-chain rehabilitation program who ultimately achieved full recovery with radiographic healing and normalized mechanics.

Rehabilitation strategies varied in scope but commonly targeted GIRD, posterior shoulder tightness, scapular mechanics, and rotator cuff strength (Table 2). Zipser *et al*. [15] described a multimodal rehabilitation program including soft-tissue mobilization, scapular stabilization, and a progressive return-to-throwing plan. Casadei and Kiel [22] advocated for interprofessional treatment involving physicians, physical therapists, coaches, parents, and nurses, recommending 3–6 months of rest followed by gradual core and rotator cuff strengthening and staged sport reintroduction once pain-free motion and symmetric strength were achieved. However, Heyworth *et al*. [11] and Jensen *et al*. [19] both found that formal physical therapy did not significantly influence recurrence risk or time to recovery.

Although conservative treatment remains the standard for treating LLS, emerging modalities have been explored to promote healing, alleviate symptoms, and facilitate return to throwing. Omodani *et al*. [21] reported successful treatment using extracorporeal shockwave therapy in two elite gymnasts who achieved rapid symptom resolution and radiographic healing within 2 months, with complete physeal closure by 7 months and sustained elite performance at 5-year follow-up.

Short-term outcomes across all studies were highly favorable, with most athletes returning to sport [11, 14, 18, 20, 21]. Fehr *et al*. [18] demonstrated that reductions in physeal widening on US correlated with improvement in resting pain, supporting the utility of imaging in guiding rehabilitation. However, recurrence was a meaningful concern, with recurrence rates ranging from 7% to 35% across studies, occurring at a mean of 6–9 months after return to sport [12, 14, 18]. Jensen *et al*. [19] reported that recurrent athletes were substantially less likely to continue baseball at long-term follow-up. Harada *et al*. [14] identified multiple risk factors for recurrence, including inadequate early rest, persistent early symptoms, and restricted internal rotation.

Long-term sequelae were also noted. Ito *et al*. [16] showed that athletes with prior LLS had significantly higher rates of subsequent shoulder (80%) and elbow pain (70%) in high school compared with controls. Radiographic healing was generally reliable, though rare complications were reported. Uchino *et al*. [24] demonstrated that resisted internal and external rotation testing to guide return-to-sport decisions produced favorable outcomes and aligned with imaging improvement.

### Injury prevention

Several studies identified modifiable participation factors that increase injury risk, including year-round play, multi-team participation, and single-sport specialization [23]. Fortney *et al*. [23] highlighted that early sport specialization, particularly without structured injury-prevention guidelines, may predispose athletes to later shoulder pathology. Shoulder pain in youth baseball players has also been found to be associated with risk factors such as strength imbalances and loss of shoulder range of motion [11, 13].

Recommended injury prevention strategies emphasize adequate rest, biomechanical training, and avoidance of early sports specialization [1, 2, 6]. Proper pitching mechanics, adherence to age-based pitch count limits, and taking at least one season off from throwing each year are believed to reduce the risk of LLS [9, 17]. The USA Baseball Medical & Safety Advisory Committee supports pitch count monitoring and mandated rest days to guide safe participation [17]. However, although pitch count enforcement and mandatory rest periods are widely recommended by consensus guidelines [2, 3], they have not been prospectively validated for LLS prevention.

While no study has directly evaluated prevention programs for their effectiveness in reducing LLS incidence specifically, evidence from general throwing injury prevention trials supports the role of comprehensive warm-up programs, posterior shoulder stretching, and external rotation strengthening in reducing overall upper extremity throwing injuries in youth baseball players [3, 6]. Collectively, the evidence supports a multifactorial prevention approach centered on workload regulation, enforced rest periods, correction of biomechanics, neuromuscular conditioning, and delayed sport specialization to reduce physeal stress injuries in youth overhead athletes.

## Discussion

This systematic review analyzed the last decade of evidence on physeal stress injuries involving the epiphyseal growth plate complex of the shoulder in young athletes. Studies have shown that there is a greater awareness of LLS, as evidenced by the increasing reported cases over time and higher rates of advanced imaging findings in the highest risk young athletes [5, 10, 11]. However, the prevalence of LLS remains challenging to quantify due to varied diagnostic criteria, underreporting of symptoms due to concern of being removed from play, and inconsistent imaging findings. Several studies have reported on the prevalence of LLS among young baseball players; however, these cross-sectional estimates have important methodological limitations. Because data are collected at a single time point, prevalence figures cannot distinguish between acute injuries, chronic or healing conditions, and incidental imaging findings in asymptomatic athletes. With MRI abnormalities in asymptomatic Little League players, imaging-based prevalence may substantially overestimate clinically significant disease. Furthermore, cross-sectional studies are subject to survival bias, as athletes who developed LLS and subsequently discontinued baseball are not captured, potentially underestimating the true burden of injury.

Critically, no studies to date have reported true incidence rates of LLS. All existing epidemiological data on LLS are limited to prevalence estimates or case series without exposure denominators. This absence of incidence data represents a fundamental gap in the literature, as incidence rates are essential for quantifying injury risk, comparing risk across age groups and competition levels, and evaluating the effectiveness of preventive interventions over time. Establishing prospective injury surveillance systems that capture both LLS diagnoses and detailed exposure data should be a priority for future research. Without such data, the true scope of LLS and the impact of current prevention strategies remain unknown.

The recent literature shows clear areas of agreement including typical demographics, regular use of plain radiographs, effectiveness of rest-based treatment, and multifactorial prevention strategies [12, 14]. Important areas of controversy include the optimal rest duration and return-to-play criteria, the role of formal physical therapy, the significance of structural remodeling, and the need for standardized diagnostic and outcome measures [12, 18, 23, 26].

LLS is primarily described in male, skeletally immature overhead athletes, with youth baseball players being the highest-risk group. Although baseball remains the dominant sport, the extension of LLS to athletes in tennis, volleyball, gymnastics, swimming, and cricket suggests that physeal stress injuries of the proximal humerus are not exclusive to throwing sports [15, 21]. This broader sports distribution reinforces that the underlying mechanism is not merely baseball-specific but rather related to repetitive physeal loading in skeletally immature athletes.

Mean ages at diagnosis are reported to be between 11 and 15 years, corresponding to the period of an open proximal humeral physis and peak height velocity. Although the physis is more vulnerable to injury during the adolescent growth spurt, there have been no studies examining the relationship between peak height velocity and the development of LLS in baseball players [26]. The most common physical examination presentation reported included tenderness over the proximal humeral physis, internal rotation deficits, scapular dyskinesis, and kinetic-chain dysfunction [12]. The reviewed literature uniformly supports standard shoulder radiographs as the first-line and often sufficient for diagnosis [12, 14]. Comparison views of the contralateral shoulder are widely recommended to evaluate subtle differences in widening, irregularity, and/or sclerosis [11]. However, the expanding roles of MRI, CT, and US in detecting physeal abnormalities and guiding clinical management of LLS remain incompletely defined [11, 14, 21].

All recent studies advocate initial cessation of throwing as the cornerstone of treatment. While durations vary, there is consensus that 2–4 months of rest from throwing is usually required, with symptom resolution typically occurring within 2–3 months and return to sport around 3–4 months, if pain has resolved and strength and motion are restored [12, 16, 17, 23]. Formal physical therapy is broadly viewed as beneficial, particularly to address GIRD, posterior shoulder tightness, scapular dyskinesis, and weakness. However, multiple studies report similar outcomes between structured physical therapy and home programs, leading to the agreement that adequate rest and a gradual symptom-guided return are key, rather than a specific rehabilitation protocol [12, 18] and a few studies even showed no difference in recurrence or recovery outcomes when comparing rest alone versus physical therapy [10, 11].

Prevention strategies include adherence to age-based pitch counts and mandated rest days, avoidance of year-round throwing and early single-sport specialization, monitoring and correction of shoulder range-of-motion and strength deficits, emphasis on proper throwing mechanics, and use of structured interval throwing programs after injury [9–13, 15, 16, 19, 24]. Of note, there is evidence that prevention strategies such as pitch count guidelines do not effectively eliminate LLS and shoulder pain [5, 11], suggesting that limiting pitch counts alone may not be sufficient for reducing injury rates.

While current injury prevention strategies for LLS primarily involve risk avoidance, Karasuyama *et al* [5, 6] highlighted that active interventional programs may be beneficial in addressing modifiable biomechanical deficits. The latter study reviewed research performed by Sakata *et al*. including a prospective cohort study and a randomized controlled trial that showed structured warm-up programs incorporating stretching, strengthening, dynamic mobility, and balance exercises significantly reduced the incidence of throwing-related shoulder and elbow injuries in youth baseball players aged 8–11 years [27, 28]. These programs addressed GIRD, posterior shoulder tightness, hip range of motion, scapular muscle strength, and thoracic kyphosis. While these studies did not specifically isolate LLS as an outcome, the biomechanical deficits addressed are directly relevant to the pathology seen in proximal humeral physeal stress. Future research should evaluate whether active interventional programs such as shoulder flexibility, glenohumeral arc of rotation, and rotator cuff strength can specifically reduce the incidence of LLS, as current prevention recommendations remain largely limited to workload management.

The severity of LLS is generally characterized as a condition with excellent short-term prognosis and low risk for premature physeal closure or long-term growth disturbance [15]. However, there is a wide variation in recurrence rates (7%–25% short term and 41% long term) [9, 11]. Specifically, long-term data have shown that ~40% may experience recurrent symptoms within 5 years, with only about half continuing baseball participation [14]. Additionally, complications from LLS are rare, but recent studies suggest that humeral remodeling may have long-term implications [12, 21]. The clinical significance of these changes is unknown. Prospective cohort studies are needed to better understand the severity of LLS, characterize associated humeral remodeling, and identify factors influencing radiographic progression, clinical outcomes, and future sport participation.

Despite several controversies that this systematic review has outlined, there have been several important areas of growth in our understanding of LLS. Advanced imaging techniques have shown changes within the proximal humerus, such as humeral retrotorsion and varus changes [12, 21]. This suggests that LLS is far more complex than a physeal injury. There is no new formalized definition of LLS, but the traditional definition of LLS focusing solely on proximal humeral physeal widening is likely incomplete. With the new findings, expanding the definition to encompass a more inclusive spectrum of osseous pathology resulting from repetitive throwing stress on the immature skeleton could be considered.

While this systematic review highlights the most recently published studies on LLS, it also brings forth the need for ongoing, high-quality, research studies. There is a significant need for prospective, large-scale, multi-center studies to uncover the true incidence and progression of LLS within the youth athlete population investigating how pitch count compliance, delayed sports specialization, adherence to rest recommendations reduce injury rates. Additionally, these studies need to help standardize diagnostic criteria utilizing clinical and imaging findings for LLS, standardize physical therapy treatments, and develop structured protocols on return-to-throw and return-to-sport criteria [10, 11]. Long-term studies are needed to evaluate the overall effect of humeral retrotorsion changes and whether this contributes to long-term morbidity [12, 21]. A classification system needs to be developed to help define injury severity and prognostic indicators with regard to imaging findings, symptom duration and functional limitations. Identifying those predictors of recurrence (i.e.: physeal widening on plain radiographs) would be helpful in counseling young athletes with LLS.

## Conclusion

LLS continues to be a common overuse condition of the shoulder involving injury to the proximal humeral physis of skeletally immature overhead young athletes. It affects primarily young male athletes who participate in overhead sports, such as baseball, with year-round, multi-team participation, and early sports specialization. Although LLS is primarily a clinical diagnosis, plain radiographs can be a helpful imaging modality.

The true prevalence and injury rates of LLS are poorly understood, likely due to underreporting; however, overall awareness of the condition has been improving. Short-term outcomes are excellent with the treatment of LLS, which involves a combination of a period of rest and rehabilitation prior to returning to sport. Long-term outcomes are still unclear, with some studies showing anatomic changes of the proximal humerus. Prospective, multi-center studies are needed to identify the optimal treatment for LLS, effective prevention strategies, and overall injury rates.

## Data Availability

No new data were created or analyzed in this study. Data sharing is not applicable to this article.
